# Paediatric Preoperative Fasting; But How Long?

**DOI:** 10.5152/TJAR.2022.2806

**Published:** 2022-06-01

**Authors:** Z. Serpil Ustalar Özgen

**Affiliations:** 1 Department of Anaesthesiology and Reanimation, University of MAA, İstanbul, Turkey

In this issue, the article ‘The Preoperative Fasting Conundrum:An Audit of Practice in a Tertiary Care Children’s Hospital’ by Sujata Shivlal Rawlani, Nandini Malay Dave, Priyanka Pradeep Karnik is published. The authors aim to study preoperative fasting times of children undergoing elective surgeries and to analyze the compliance with current guidelines. The audit in their study had been undertaken to reveal the compliance and the effect of education on the duration of preoperative fasting in their hospital. Some interventions made to promote compliance with current fasting guidelines. A detailed preoperative history was obtained regarding the time of the last intake of solids, breast milk, formula milk, juices, and clear liquids by the child. In addition, the source from whom the preoperative fasting orders were received, and details of exact instructions were noted. The preoperative fasting time for food (solids and milk) ranged between 2 hours and 15 hours (mean ± SD 9.43 ± 2.89 hours). The preoperative fasting time for clear liquids ranged between 2 hours and 10.5 hours (mean ± SD 6.64 ± 2.12 hours). They had mentioned that many of the children were kept fasting overnight irrespective of the timing of the surgery. The fasting guidelines they had used was the 6-4-2 regimen. The article had been accepted for publication on September 16, 2021. 

In Eur J Anaesthesiol 2022; 39:4–25, Peter Frykholm, Nicola Disma, Hanna Andersson, Christiane Beck, Lionel Bouvet, Eloise Cercueil, Elizabeth Elliott, Jan Hofmann, Rebecca Isserman, Anna Klaucane, Fabian Kuhn, Mathilde de Queiroz Siqueira, David Rosen, Diana Rudolph, Alexander R. Schmidt, Achim Schmitz, Daniel Stocki, Robert Sumpelmann, Paul A. Stricker, Mark Thomas, Francis Veyckemans and Arash Afshari had published a ‘A guideline from the European Society of Anaesthesiology and Intensive Care on the Preoperative Fasting in Children. They had stated that conservative preoperative fasting regimes had been recommended for many years and in the recent publications employing more liberal fasting regimes, no increase in rates of regurtitation or aspiration had been reported. Fasting times of 6 h for solid food and formula, 3-4 h for breast milk and 2 h for clear fluid were commonly advocated in most of the literature, these protocols having good safety profile, carry the risk of excessive fasting times, thirst, distress, besides, dehydration, hypoglycemia, metabolic imbalances. 

In this guideline at 2022, it is strongly recommended that prolonged fasting times should be avoided in all children whenever possible, since this may cause ketone body accumulation, low systolic blood pressure during induction. It is also recommended that healthy children should be encouraged to drink clear fluids (clearly defined to caregivers, including water with or without sugar, pulp-free juice and milk free tea or coffee) up to 1 h before aneaesthesia induction for elective procedures (1C) It is also recommended that for infants, breast milk feeding should be encouraged until 3 h before anaesthesia induction. (1C) For infants, formula (or nonhuman) milk may be encouraged until 4 h before anaesthesia induction. (2B) Solid food should be allowed until 6 h before anaesthesia induction. A light breakfast of solids or nonclear fluids may be allowed up to 4 h prior to anaesthesia induction. 

For healthy children, the authors conclude that the new 6-4-3-1 regimen (6 h for solids, 4 h for formula and nonhuman milk, 3 h for breast milk, 1 h for clear fluids) can be safely recommended. This article in our journal had used the previous guideline, however despite the guidelines, it is obvious that, we tend to fast our children more than necessary. The list of suggestions and and findings, in this article and the recent guidelines makes it critical to revise and develop institutionally approved new fasting protocols, including ‘special’ cases, and closely watch their application. This could be achieved only with close supervision, clear guidelines, education for all parts of the process regarding the organization of fasting, from caregivers, nurses, surgeons, as well as anaesthesia workers. 

## References

[1] Frykholm P Disma N Andersson H et al. Pre-operative fasting in children a guideline from the European Society of Anaesthesiology and Intensive Care. Eur J Anaesthesiol. 2022;39(1):4 25. 10.1097/EJA.0000000000001599) 34857683

[2] Rawlani SS Dave NM Karnik PP . The preoperative fasting conundrum: an audit of practice in a tertiary care Children’s Hospital. Turk J Anaesthesiol Reanim. 2022;50(3):207 211. 35801327 10.5152/TJAR.2022.21132 PMC9361398

[3] Frykholm P Schindler E Sümpelmann R Walker R Weiss M . Preoperative fasting in children: review of existing guidelines and recent developments. Br J Anaesth. 2018;120(3):469 474. 10.1016/j.bja.2017.11.080) 29452803

[4] Engelhardt T Wilson G Horne L Weiss M Schmitz A . Are you hungry? Are you thirsty? Fasting times in elective out­patient pediatric patients. Paediatr Anaesth. 2011;21(9):964 968. 10.1111/j.1460-9592.2011.03573.x) 21489044

[5] Williams C Johnson PA Guzzetta CE et al. Pediatric fasting times before surgical and radiologic procedures: benchmarking institutional practices against national standards. J Pediatr Nurs. 2014;29(3):258 267. 10.1016/j.pedn.2013.11.011) 24365219

[6] Al-Robeye AM Barnard AN Bew S . Thirsty work: exploring children’s experiences of preoperative fasting. Paediatr Anaesth. 2020;30(1):43 49. 10.1111/pan.13759) 31665824

[7] Andersson H Zarén B Frykholm P . Low incidence of pulmonary aspiration in children allowed intake of clear fluids until called to the operating suite. Paediatr Anaesth. 2015;25(8):770 777. 10.1111/pan.12667) 25940831

